# Docking site interventions following bone transport using external fixation: a systematic review of the literature

**DOI:** 10.1007/s00264-023-06062-8

**Published:** 2023-12-27

**Authors:** E. Liodakis, V. P. Giannoudis, P.J. Harwood, P. V. Giannoudis

**Affiliations:** 1 Department of Trauma Surgery, Hannover Medical School, Carl-Neuberg-Str. 1, 30625 Hannover, Germany; 2 Academic Department of Trauma & Orthopaedics, School of Medicine, University of Leeds, Leeds General Infirmary, Leeds, UK

**Keywords:** Docking site, Bone transport, Non-union, Bone defect, Open fractures

## Abstract

**Purpose:**

Although bone transport is a well-recognised technique to address segmental bone defects, optimal management of docking sites is not absolutely determined. Some surgeons routinely intervene in all cases, and others prefer to observe and intervene only if spontaneous union does not occur. Primary aim of the study was to compare rates of docking site union between patients who underwent routine docking site intervention and those who did not.

**Methods:**

A systematic literature review using the keywords “bone transport”, “docking”, “tibia”, and “femur” was performed in PubMed using PRISMA guidelines. Studies published in English from January 2000 to August 2022 were included and assessed independently by two reviewers. Pooled analysis was undertaken dividing patients into two groups: those managed by routine intervention and those initially observed.

**Results:**

Twenty-three clinical studies met the eligibility criteria for pooled analysis, including 1153 patients, 407 in the routine intervention and 746 in the observed group. The rate of union after initial treatment was 90% in the routine intervention group and 66% in the observed group (*p* < 0.0001). Overall union rates at the end of treatment were similar at 99% in both groups. Patients in the observed group required an average of 2.2 procedures to achieve union overall compared with 3.8 in the routine intervention group. Time in frame was similar between groups.

**Conclusion:**

Based on the current literature, routine docking site interventions cannot be recommended, since this may lead to unnecessary interventions in two thirds of patients. Timely selective intervention in those at high risk or after a defined period of observation would appear to be a logical approach.

## Introduction

Critical segmental bone defects present one of the most challenging situations faced by orthopaedic surgeons. Although the Masquelet technique has risen in popularity, perhaps related to the familiarity of required implants and techniques [1–4], distraction osteogenesis remains the most widely utilised approach for bone regeneration [5, 6]. Popularised by Ilizarov, the technique employs callus distraction to form new bone [7]. An osteotomy is undertaken away from the defect, and after a short latent period to allow callus formation to begin, gradual distraction is applied, traditionally by external fixation, at a rate of around 1 mm a day. The main purported advantages of this over other methods include elimination of donor site morbidity from bone graft harvest, avoidance of internal implants (particularly in cases complicated by infection), reduced violation of the soft tissue envelope, early or immediate weight bearing and the creation of a regenerate bone similar in diameter and morphology to healthy long bones [7, 8]. It has also been suggested that the increased limb blood flow generated during distraction may be helpful in eliminating infection [9], making its use in defects resulting from open fractures, fracture-related infections or osteomyelitis particularly attractive [10].

**Fig. 1:**
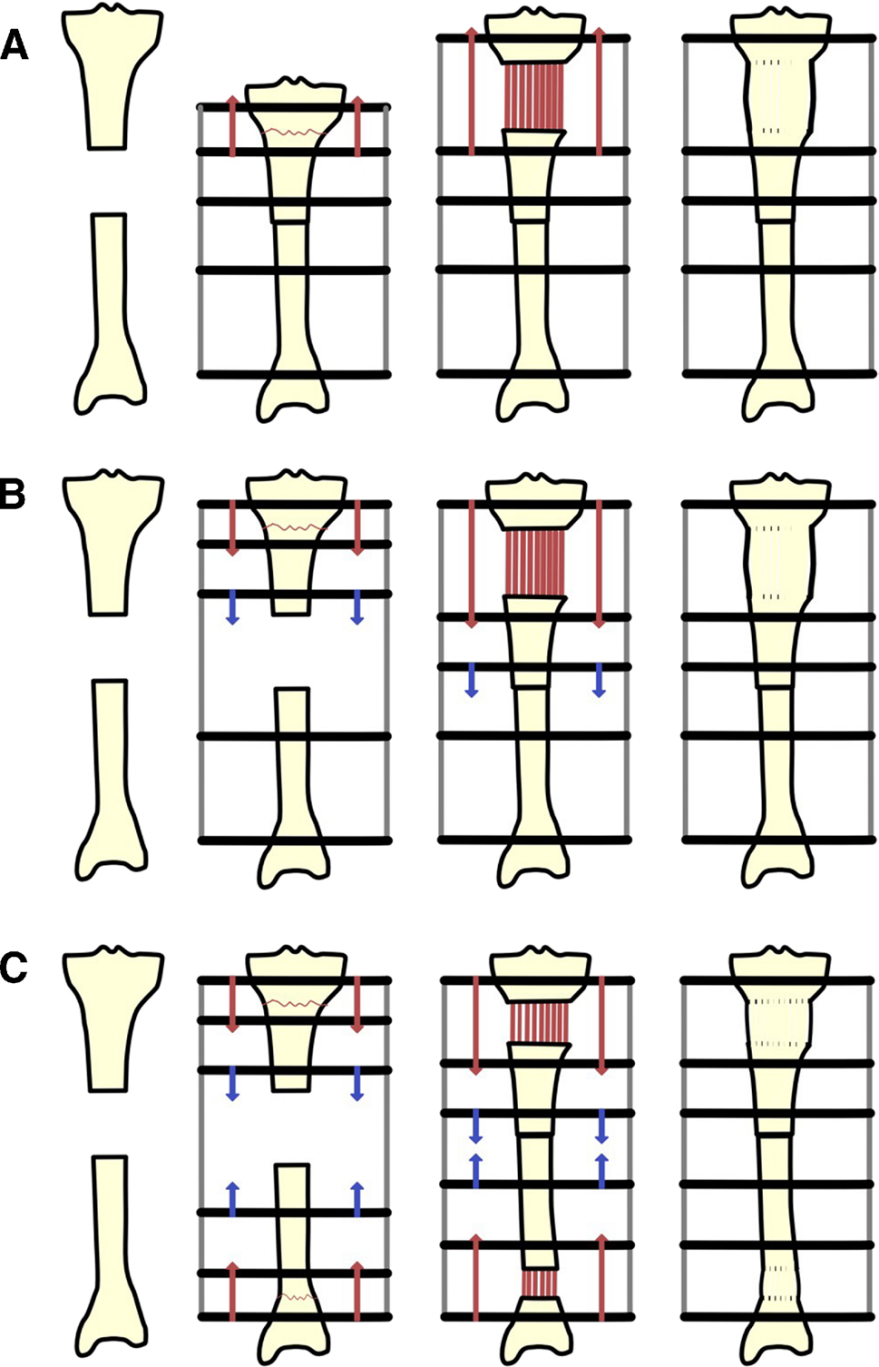
Demonstration of the docking site (*) for the different bone transport techniques. **A** First shortening then lengthening, **B** bifocal bone transport, **C** trifocal bone transport

There are a variety of strategies which can be used when treating osseous defects by bone transport. These can be defined based on the initial management of the bone defect and the number of distraction sites. In shorter defects, usually 5 cm or less, the gap can be acutely shortened to achieve immediate contact, and then, the bone lengthened at a distant site. This is termed acute shortening and re-lengthening [11]. Maintaining overall length and moving a segment of bone to close the defect over time are known as bone transport. This can be bifocal, with a single osteotomy site for distraction, or trifocal where a double osteotomy is used to create two distraction sites (Fig. 1) [12, 13].

Distraction osteogenesis can be achieved using circular or monolateral fixators [12, 14, 15] or more recently using magnetic nails for all internal transport [16–18]. Combination with internal fixation can be undertaken to reduce external fixator time [19–21]. There are also reports of utilising membrane induction as in the Masquelet technique in an attempt to improve healing [22]. Regardless of technique, the result is the apposition of the two opposing surfaces of the bone defect at the “docking site”. Achieving union at the docking site can be challenging. Opinion varies regarding the management of these sites, with some surgeons choosing to simply apply ongoing compression for a period and others undertaking planned surgical interventions at the time of docking to augment healing. Various strategies to enhance union have been described, including open [12, 23], closed [8, 24] and endoscopic techniques[25], the use of bone grafts or growth factors [12, 25–27] and the application of additional implants. This does however risk further violation of an already traumatised soft tissue envelope in the presence of external fixation hardware, which makes the maintenance of sterility during the procedure and access more difficult. It therefore potentially places the patients at risk of catastrophic complications which could jeopardise the treatment outcome. There is no generally accepted consensus, and docking site protocols remain a controversial topic [27–29]. We therefore undertook a systematic review of the literature regarding this topic with the aim of answering the following questions:

In adult patients undergoing distraction osteogenesis for lower limb bone defects resulting from trauma, osteomyelitis or fracture-related infection, compared to simple compression, is planned operative docking site intervention associated with

1. Increased union rates without further intervention?
2. Increased overall treatment failure?

As a secondary aim, we planned to examine the literature in an attempt to determine which bone transport strategies/locations were associated with increased docking site union.

## Methods

### Literature search strategy

**Fig. 2:**
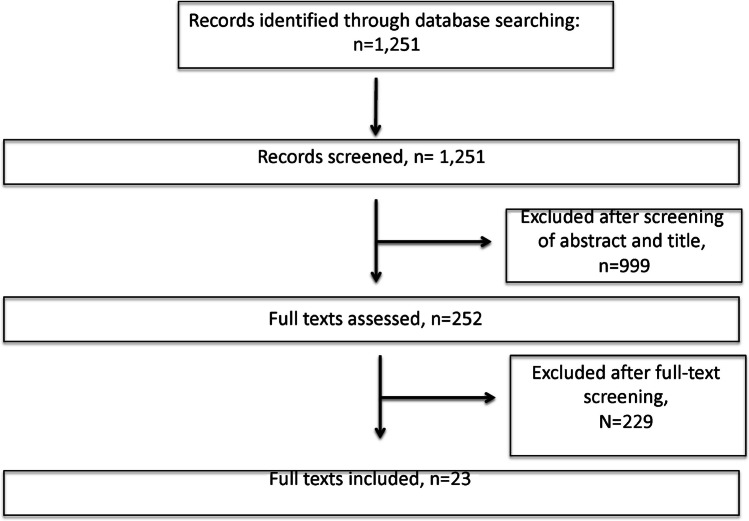
Flowchart demonstrating literature search strategy

A systematic search according to the Preferred Reporting Items for Systematic Reviews and Meta-Analysis (PRISMA) statement [30] using the search terms “((bone transport) OR (docking)) AND ((tibia) OR (femur))” was performed. The search was limited to the database Medline. During initial screening, articles, titles and abstracts were reviewed by the first and second authors for relevance. Relevant full-text articles were then obtained and evaluated according to the eligibility criteria of the study and relevant data extracted (Fig. 2). Any disagreement between the two reviewers was resolved by consensus following the discussion with the senior author. The reviewers were not blinded to the names of authors, institutions or journals.

### Eligibility criteria

Studies were included if they met the following criteria:

1. Bone transport is performed by external fixation for bone defects resulting from trauma, osteomyelitis or fracture-related infections of the lower extremity
2. Reported outcomes regarding the healing of the docking site
3. Clinical studies in humans with the full-text paper published between January 2000 and August 2022

Studies were excluded if they met the following criteria:

1. Reviews, conference abstracts, animal studies, letters or comments
2. Full-text paper written in English was not available
3. Studies which routinely combined internal with external fixation
4. Studies where the reporting precluded accurate data extraction for the main review outcomes
5. Studies including less than 10 patients

### Assimilation of data

Studies were divided into groups depending on whether docking sites underwent planned operative intervention (planned intervention) of any nature or were observed initially (observed), and intervention is undertaken only if union did not occur. Where studies included patients managed by both approaches, these were reported in each group as appropriate. The main study outcomes were the number of patients in whom uncomplicated docking site union occurred (no further interventions to union, no docking site refracture after frame removal), the mean number of procedures to achieve union and the number of patients in whom union was ultimately achieved. Other data extracted from the manuscripts included the author’s details, sample size, location of bone defect, specific inclusion/exclusion criteria, fixation device, type of osteotomy, soft tissue coverage requirements, docking procedures, size of defect, healing index, external fixator time and mean bone transport time. A narrative literature review summarising relevant findings from individual papers, particularly focused on risk factors for docking site non-union, is presented alongside the pooled analysis. Studies excluded from the pooled analysis were still included in the narrative review where relevant.

### Statistical analysis

Insufficient prospective comparative studies were identified to allow formal meta-analysis of randomised controlled trials. Extracted data were therefore described as in the original manuscripts, and pooled analysis of extracted data from observational studies was undertaken to answer the 2 main research questions. A chi-squared or Fisher’s exact test was used as appropriate to compare nominal results. Statistical analysis was undertaken using GraphPad computer software (Version 6.04).

## Results

### Does routine docking site intervention improve outcomes?

The initial search identified 1251 publications. Twenty-three of these met the eligibility criteria for the pooled analysis, including 1153 patients treated by distraction osteogenesis [8, 12, 14, 15, 22–28, 31–42]. Details of each study are shown in Table 1. Extracted data from each study and pooled analysis are shown in by docking site intervention group in Table 2 (planned intervention) [12, 14, 15, 23, 25–28, 31–33] and Table 3 (observed) [8, 24, 33, 38–42]. Where patients from a single study had different docking site protocols, these were included in the relevant group. In total, there were 407 patients in the planned intervention group and 746 in the observed group. In the planned intervention group, 368 of 407 (90%) docking sites went on to union without further intervention, compared to 497 of 746 (66%) in the observed group. This result was statistically significant (chi-squared *p* < 0.0001). Included within these failures at the docking site were 7 refractures in the planned intervention group [12, 28, 32, 33] compared with 21 in the observation group [23, 24, 27, 32–34, 41] (not significant, *p* = 0.28). For those studies reporting the outcome, more surgical procedures overall (including planned and unplanned) were required to achieve union in the planned intervention group than the observation group (mean 3.8 in 160 patients from four studies vs. mean 2.2 in 333 patients from 10 studies). The proportion of patients in whom union was ultimately achieved was similar between the two groups, being 402 of 407 (99%) in the planned procedure group vs. 737 of 746 (99%) in the observed group (chi-squared *p* =0.612). Overall treatment time is difficult to estimate between groups without access to the raw data. Calculating an average based upon the data as reported in each paper, where this was available, and the number of patients treated reveals that this is similar at 383 days (275 patients) in the planned intervention group and 393 days (642 patients) in the observed group. These results were highly variable between studies due to their heterogenous nature, and the methodology of pooled analysis is weak; these results should be viewed with caution.

**Table 1:** Summary of papers included in the pooled analysis

Author	Year	Type of transport	Sample size	Bone affected	Fixation device	Reason for bone defect	Flap	Docking procedure	Immediate docking procedure (yes/no)	Graft at docking site	Growth factors at docking
Catagni et al. [12]	2019	Bifocal/trifocal	86	Tibia	Ilizarov frame	Open fractures/infected non-unions/osteomyelitis	0	86	Yes	22 (If bone ends sclerotic or insufficient bone contact)	0
		Bifocal	45	Tibia	Ilizarov frame	As above	0	45	Yes	12	0
		Trifocal	41	Tibia	Ilizarov frame	As above	0	41	Yes	10	0
El-Alfy [14]	2017	Bifocal	28	Tibia Proximal 1/3 *n* = 6 Middle 1/3 *n* = 13 Distal 1/3 *n* = 9	Ilizarov frame	Open fractures, infected, non-union osteomyelitis	2	15	Yes	15	0
Lovisetti et al. [23]	2013	Bifocal/trifocal	45	Tibia	Ring fixators	As above	0	29	Yes & no	0	0
		Group A—single compression docking site technique	12 (Bifocal)	Tibia	Ilizarov frame (*n =* 10), TrueLok (*n =* 4), TSF (*n =* 2)	Local Infection present in 11/16 cases (69%)	0	0	No	0	0
			4 (Trifocal)					0	No	0	
		Group B—open docking site technique	9 (Bifocal)	Tibia	TSF (*n =* 18), Sheffield external fixator (*n =* 3), true lock (*n =* 2)	Local infection was present in 18 of 23 cases (78%)	0	23	Yes	23 (autologous bone grafting)	0
			14 (Trifocal)						Yes		
		Group C—endoscopic docking technique	6 (Bifocal)	Tibia	Truelok (*N =* 3), TSF (*N =* 3)	Not stated	0	6	Yes	0	0
Miraj et al. [31]	2021	Bifocal	14	Tibia	Ilizarov frame	Infected non-unions	0	14	Yes	0	0
Sala et al. [25]	2013	Bifocal	27	Tibia	Circular ring fixator (TSF, Truelok, Sheffield)	Segmental tibia loss, atrophic non-union or resection of osteomyelitic bone	0	27	Yes	0	0
		Group A—endoscopic docking site	9	Tibia	TSF (*n =* 6), true lock (*n =* 3)	As above	0	9	Yes	0	0
		Group B—standard open grafting of docking site	18	Tibia	TSF (*n =* 14), Sheffield frame (*n =* 3), TLK (*n =* 1)	As above	0	18	Yes	18	0
Hatzokos et al. [32]	2011	Bifocal	43	Tibia	Monolateral frames (*n =* 34), circular frame (*n =* 4), hybrid fixation (*n =* 5)	Open fractures/infected non-union/osteomyelitis/tumour	0	32	Yes/no	22	10
		Group A—closed compression group	11	Tibia	Not listed per subgroup	Septic Pseudoarthrosis (*n =* 8), osteomyelitis (*n =* 2), tumour resection (*n =* 1)	0	0	No	0	0
		Group B—surgical debridement of interposed tissue and application of autologous bone graft prior to docking	22	Tibia	Not listed per subgroup	Septic pseudoarthrosis (*n =* 13), osteomyelitis (*n =* 5), trauma (*n =* 4)	0	22	Yes	22—Corticocancellous autologous iliac bone grafting	0
		Group C—surgical debridement of docking site through application of DBM and centrifuged autologous bone marrow aspirate	10	Tibia	Not listed per subgroup	Septic pseudoarthrosis (*n =* 7), osteomyelitis (*n =* 2), trauma (*n =* 1)	0	10	Yes	0	10 (iliac crest bone marrow aspirate mixed with DBM)
Lovisetti and Sala [27]	2011	Bifocal/trifocal	31	Tibia		Infected non-unions (*n =* 23), trauma (*n =* 8)	0	18	Yes/no	0	0
		Group A—docking site was compressed after bone transport was complete, without removal of the interposed tissue	11 (Bifocal)	Tibia	Ilizarov frame (*n =* 10), TrueLok (*n =* 1), TSF (*n =* 2)	Infected non-unions *n =* 9, trauma *n =* 4	0	0	No	0	0
			2 (Trifocal)								
		Group B—docking site procedure included a routine “refreshing” of the bone ends	7 (Bifocal)	Tibia	TSF (*N =* 15), Ilizarov frame (*N =* 3)	Infected non-unions *n =* 14, trauma *n =* 4	0	18	Yes	0	0
			11 (Trifocal)				0				
Spiegl et al. [26]	2013	Bifocal	25	Tibia	Ilizarov (*n =* 25) *Of note, 13 had plating procedures*	Posttraumatic tibial osteitis	15 (4 local and 11 free flap	25	Yes	If no plating—11 With plating included—9	If no plating—5 With plating included—5
Kinik and Kalem [28]	2019	Bifocal	30	Tibia Proximal 1/3 (*n =* 6) Middle 1/3 (*n =* 9) Distal 1/3 (*n =* 15)	Ilizarov (*n =* 30)	Infected tibial non-unions with minimum bone loss of 6 cm after debridement	0	27	Yes/no	0	0
		Group A—closed docking	3	Not listed per subgroup	Ilizarov (*n =* 3)	Infected tibial non-unions with minimum bone loss of 6 cm after debridement	0	0	No	0	0
		Group B—open docking	27	Not listed per subgroup	Ilizarov (*n =* 27)	Infected tibial non-unions with minimum bone loss of 6 cm after debridement	0	27	Yes	0	0
Iacobellis et al. [15]	2010	Bifocal/trifocal In 5 cases (2 tibias, 3 femurs), variation of bone transport technique used through shortening by 15% and subsequent lengthening	100	Tibia/femur	Ilizarov (*N =* 55), monolateral (*N =* 45)	See below	0	100	Yes	Yes (Not clearly stated how many had graft— “If small residual gaps were seen between the two bone ends, cancellous bone taken from the ipsilateral iliac crest and grafted”)	0
		Bifocal (*n =* 63), trifocal (*n =* 10)	74	Tibia	Ilizarov (*N =* 49), monolateral (*N =* 25)	Open fracture (*n =* 13), osteomyelitis (*n =* 25), infected non-unions (*n =* 32), atrophic non-unions (*n =* 5)	0	74	Yes	Yes (Not clearly stated how many had graft— “If small residual gaps were seen between the two bone ends, cancellous bone taken from the ipsilateral iliac crest and grafted”)	0
		Bifocal (*n =* 17), trifocal (*n =* 5)	26	Femur	Monolateral (*N =* 18), Ilizarov (*N =* 8)	Open Fracture (*n =* 6), osteomyelitis (*n =* 4), infected non-unions (*n =* 13), atrophic non-unions (*n =* 2)	0	26	Yes	Yes (Not clearly stated how many had graft— “If small residual gaps were seen between the two bone ends, cancellous bone taken from the ipsilateral iliac crest and grafted”)	0
Liu et al. [34]	2020	Bifocal (*n =* 221), trifocal (61)	282	Tibia (*n =* 220) Femur (*n =* 62)	Ilizarov (*n =* 128), monolateral external fixator (*n =* 154)	Post-traumatic (*n =* 97), osteomyelitis (*n =* 146), infected non-union (*n =* 26), atrophic non-union (*n =* 13)	Yes (not clearly stated)	63 soft tissue incarceration was noted cases and managed by freshening the bone ends, opening the medullary canal and resection of invaginated soft tissue	No	Delayed union was presented in 38 cases and treated by “accordian” technique or bone grafting if developed to non-union	0
		Bifocal trifocal	220	Tibia Proximal 1/3 (*n =* 28) Middle 1/3 (*n =* 98) Distal 1/3 (*n =* 94)	Ilizarov (*n =* 128), monolateral external fixator (*n =* 92)	Post-traumatic (*n =* 80), osteomyelitis (*n =* 115), infected non-union (*n =* 15), atrophic non-union (*n =* 10)	Yes (not clearly stated)	Yes (not clearly stated)	No	Delayed union was presented in 38 cases and treated by “accordian” technique or bone grafting if developed to non-union	0
		Bifocal trifocal	62	Femur Proximal 1/3 (*n =* 4) Middle 1/3 (*n =* 31) Distal 1/3 (*n =* 27)	Monolateral external fixator (*n =* 62)	Post-traumatic (*n =* 17), osteomyelitis (*n =* 31), infected non-union (*n =* 11), atrophic non-union (*n =* 3)	Yes (not clearly stated)	Yes (not clearly stated)	No	Delayed union was presented in 38 cases and treated by “accordian” technique or bone grafting if developed to non-union	0
Sigmund et al. [35]	2020	Monfocal bifocal	47	Tibia	Ilizarov (*n =* 47)	Infected non-union, osteomyelitis	17	18	No	4	2
		Acute shortening and re-lengthening (ASR)	20	Tibia	Ilizarov (*n =* 20)	Not listed	7	3	No	0	0
		Bifocal	27	Tibia	Ilizarov (*n =* 27)	Not listed	10	15	No	4	2
Hamiti et al. [22]	2021	First stage using induced membrane followed by second stage using trifocal bone transport technique	18	Tibia Proximal 1/3 (*n =* 6) Middle 1/3 (*n =* 8) Distal 1/3 (*n =* 4)	Ilizarov (*n =* 18)	Primary osteomyelitis (*n =* 5), post-traumatic osteomyelitis (*n =* 13)	2	6 (not clearly stated as to who had accordion/bone grafting)	No	Yes (not clearly stated who had grafting)	0
Huang et al. [36]	2021	ASR/bifocal	85	Tibia	Ilizarov (*n =* 41), hybrid fixation (Ilizarov + IM nail/plate) (*n =* 44)	Acute trauma (*n =* 48), osteomyelitis (*n =* 37)	0	15	No	12	0
		Antibiotic calcium sulphate-loaded hybrid transport (ACSLHT)	44	Tibia	Hybrid fixation (Ilizarov + IM nail/plate) (*n =* 44)	Acute trauma (*n =* 25), osteomyelitis (*n =* 19)	0	Not clearly stated	No	Not Clearly stated	0
		Traditional Ilizarov bone transport (TIBT)	41	Tibia	Ilizarov (*n =* 41)	Acute trauma (*n =* 23), osteomyelitis (*n =* 18)	0	Not clearly stated	No	Not Clearly stated	0
Huang et al. [37]	2022	Bifocal bone transport/ASR	68	Tibia	Ilizarov (*n =* 68)	Acute trauma (*n =* 31), chronic osteomyelitis (*n =* 37)	Not Clearly stated	9	No	9	0
		Acute shortening and lengthening	32	Tibia	Ilizarov (*n =* 32)	Acute trauma (*n =* 15), chronic osteomyelitis (*n =* 17)	0	4	No	4	0
		Bifocal bone transport with calcium sulphate	36	Tibia	Ilizarov (*n =* 36)	Acute trauma (*n =* 16), chronic osteomyelitis (*n =* 20)	Not Clearly stated	5	No	5	0
Tetsworth et al. [33]	2017	Bone transport/acute shortening and lengthening (ASR)	42	Tibia	Ilizarov (*n =* 42)	Infected non-union (*n =* 36), previous history of deep infection (*n =* 6)	0	22	No	22	0
		Bone transport	21	Tibia	Ilizarov (*n =* 21)	Not clearly stated	0	Not clearly stated	No	Not clearly stated	0
		ASR	21	Tibia	Ilizarov (*n =* 21)	Not clearly stated	0	Not clearly stated	No	Not clearly stated	0
Eralp et al. [38]	2016	ASR/segmental bone transport group	74	Tibia	Ilizarov (*n =* 74)	Osteomyelitis (*n =* 74)	6	26	No	26	0
		Segmental bone transport group	29	Tibia	Ilizarov (*n =* 29)	Osteomyelitis (*n =* 29)	5	18	No	18	0
		ASR	45	Tibia	Ilizarov (*n =* 45)	Osteomyelitis (*n =* 45)	1	8	No	8	0
Liodakis et al. [8]	2019	Whole cohort	39	Tibia	External fixator (*n =* 21), monorail over nail (*n =* 18)	Chronic osteitis (*n =* 24), atrophic non-union (*n =* 15)	2	13	No	2	0
		Bifocal Ilizarov fixator	21	Tibia	External fixator (*n =* 21)	Chronic osteitis (*n =* 14), atrophic non-union (*n =* 7)	0	9	No	Not clearly stated	0
		Bifocal monolateral transport over IM nail (excluded from grouped analysis)	18	Tibia	Monorail (*n =* 18)	Chronic osteitis (*n =* 10), atrophic non-union (*n =* 8)	0	4	No	Not clearly stated	0
Paley and Maar [24]	2000	Bifocal/trifocal	19	Tibia	Ilizarov (*n =* 17), hybrid (*n =* 2)	Atrophic non-union (*n =* 4), malunion (*n =* 1), infected non-unions (*n =* 14)	4	10	No	7	0
		Bifocal	13	Tibia	Not stated	Not stated		Not clearly stated	No	Not stated	0
		Trifocal	6	Tibia	Not stated	Not stated		Not clearly stated	No	Not stated	0
Li et al. [39]	2020	ASR/bifocal	26	Tibia	External fixator (Orthofix)	Trauma (*n =* 26)	Yes (not clearly stated)	6	No	6	0
		Osteotomy and unilateral bone lengthening with Orthofix external fixation	13	Tibia	External fixator (Orthofix)	Trauma (*n =* 26)	Yes (not clearly stated)	4	No	4	0
		Osteotomy and bidirectional bone lengthening with Orthofix external fixation	13	Tibia	External fixator (Orthofix)	Trauma (*n =* 26)	Yes (not clearly stated)	2	No	2	0
Aktuglu et al. [40]	2016	Bifocal	24	Tibia Proximal 1/3 (*n =* 4) Middle 1/3 (*n =* 13) Distal 1/3 (*n =* 7)	Ilizarov	Open fracture (*n =* 22), failed treatment congenital pseudoarthrosis (*n =* 1), failed osteomyelitis debridement (*n =* 1)	1	5	No	0	0
Saridis et al. [41]	2006	Bifocal	13	Femur (Distal 1/3 *n =* 15)	Ilizarov	Infected non-unions	1	5	No	2	0
Blum et al. [42]	2010	Bifocal	50	Femur	Ilizarov	Infected non-unions	0	21	No	21 (Of note, 6 patients had grafting from the iliac crest at the original bone defect)	0

**Table 2:** Patient outcome and pooled analysis for those treated by protocols including routine docking site procedures (planned intervention group)

Study	Year of publication	Treatment	Patients	Defect size (mean)	Bone graft?	Docking site procedure	Docking consolidation time (mean)	EF time/healing index (mean)	Uncomplicated docking site union	Percent	Docking site complications	Refractures	Subsequent docking site procedures	United at end of treatment	Percent	Total procedures per patient
Catagni et al. [12]	**2019**	Bifocal (*n =* 45) Trifocal (*n =* 41)	86	BF 12.5 cm (median), TF 13.5 cm (median)	Some	Docking site revision, excision of soft tissue interposition, freshening of bone ends, autologous bone graft as required (10 in TF and 12 in BF groups)		BF—EF time 345 days (median), HI 44 days/cm TF—EF time 261 days (median), HI 29 days/cm	82	95%	Refracture of docking site (*n =* 4)	4	Grafting at docking Site (*n =* 4)	86	100%	4.40
El-Alfy [14]	**2017**	Bifocal	28	8 cm	Some	Debridement and refreshening of bone ends (*n =* 28), bone graft (*n =* 15)		EF time 396 (mean)	28	100%	Nil reported at docking site.	0	Nil	28	100%	2.07
Lovisetti et al. [23]	**2013**	Group B—open docking, site technique (*n =* 23), & group C—endoscopic docking technique (*n =* 6)	29	B—9.5 cm, C—8.6 cm	Some	Debridement and refreshening of bone ends (*n =* 22), bone graft (*n =* 21), debridement and refreshening of bone ends with graft (*n =* 6)	335 days	EF time 456 days (mean)	28	97%	Non-union docking site (*n =* 1)	0	Ilizarov monofocal technique (*n =* 1)	28	97%	
Miraj et al. [31]	**2021**	Bifocal	14	14 cm	No	Debridement and refreshening of bone ends (*n =* 14)	150 days	EF Time 261.5 days	14	100%	Nil reported.	0	Nil	14	100%	
Sala et al. [25]	**2013**	Endoscopic docking site (*n =* 9), open grafting docking site (*n =* 18)	27	9.3 cm	Some	Minimally invasive freshening of bone ends & bone graft (*n =* 9), open debridement of bone ends and bone graft (*n =* 18)	133 days	Endoscopic—EF time 464 days, HI 52.9 days/cm Open—EF time 461 days, HI 48.3 days/cm	26	96%	Infected docking site in endoscopic group (*n =* 1)	0	Application of new frame & grafting at docking site (*n =* 1)	27	100%	
Hatzokos et al. [32]	**2011**	Debridement and bone graft (*n =* 22), with additional DBM and BMAC (*n =* 10)	32	9.3 cm	Yes	Debridement and application of bone graft prior to docking (*n =* 22), with additional application of DBM and autologous bone marrow aspirate (*n =* 10)	222 days, 177 days in BMAC/DBM group		27	84%	Docking site fracture (*n =* 1), non-union (*n =* 5)	1	Bone graft to docking site (*n =* 5)	32	100%	
Lovisetti and Sala [27]	**2012**	Group B—docking site procedure included a routine “refreshing” of the bone ends (*n =* 18)	18	9.4 cm	No	Debridement and refreshening of bone ends (*n =* 18)		EF time 455.6 days, HI 52.6 days/cm	18	100%	0			18	100%	
Spiegl et al. [26]	**2013**	Bifocal	25	5.7 cm	Yes	Exploration, debridement of bone ends, autologous bone graft (25), internal fixation or malalignment (4)		EF time 455.6 days, HI 52.6 days/cm	17	68%	Recurrent infection (*n =* 7), delayed Union (*n =* 6)	0	Debridement and antibiotics (*n =* 4), segmental resection and further transport (*n =* 2), amputation (*n =* 1), exogen (*n =* 5), intramedullary nail (*n =* 1)	24	96%	4.48
Kinik and Kalem [28]	**2021**	Group B—open docking (*n =* 27)	27	8.1 cm (mean)	No	Removal of the skin, invagination—if any—refreshment of the bone ends, re-opening of the medullary canal and acute compression, and reduction of the bone ends including ankle arthrodesis patients		EF time 416.7 days, HI 45.3 days/cm	26	96%	Refracture (*n =* 1)	1	Intramedullar nailing	27	100%	
Iacobellis et al. [15]	**2010**	Bifocal (80)/trifocal (20) transport, 74 tibial, 49 circular, 25 monolateral, 26 femoral, 18 monolateral, 8 circular	100	8.4 cm	Some	Debridement of docking site, removal of interposed tissue, bone graft if apposition poor. IMN in 4 femoral cases as poor regenerate		N/A	82	82%	Docking site non-union (*n =* 17), 10 femur, 7 tibia, recurrent infection resistant to further treatment (*n =* 1)	0	Decortication (*n =* 15), IMN (*n =* 2), amputation (*n =* 1)	97	97%	
Tetsworth et al. [33]	**2017**	Bifocal bone transport	21	7.0cm	Yes	Refresh and bone graft (14)	295 days (mean)	EF time 380 days, HI 54.7 days/cm (mean)	20	95%	Refracture of docking site (*n =* 1)	1		21	100%	3.00
Pooled			407					383 days	368	90%	7			402	99%

**Table 3:** Patient outcome and pooled analysis for those treated by protocols including initial period of observation with intervention only if spontaneous docking site union did not occur (observed group)

Study	Year of publication	Treatment	Patients	Defect size (mean)	Docking consolidation time (mean)	EF time/healing index (mean)	Uncomplicated docking site	Percent	Docking site complication union	Refractures	Subsequent docking site procedures	United at end of treatment	Percent	Total procedures per patient
Lovisetti et al. [23]	2013	Group A—simple docking site compression	16	6.4 cm	272 days	EF time 359 days	14	88%	Refracture of docking site (*n =* 2)	2	Further circular frame	15	94%	
Hatzokos et al. [32]	2011	Closed compression group (*n =* 11)	11	9.45 cm	365 days	N/R	8	73%	Docking Site fracture (*n =* 1), non-Union (*n =* 2)	1	Bone grafting (*n =* 2)	11	100%	
Lovisetti and Sala [27]	2012	Group A—docking site was compressed after bone transport was complete, without removal of the interposed tissue (*n =* 13)	13	6.5 cm		EF time 380 days HI 62.1 days/cm	11	85%	Docking site fracture (*n =* 1), non-union (*n =* 1)	1	Ilizarov frame to refracture, management of docking site non-union unclear	12	92%	
Kinik and Kalem [28]	2021	Group A—closed compression (*n =* 3)	3	8.1 cm	N/A	EF time 416.7 days (mean), HI 45.3 days/cm (mean)	2	67%	Non-union (*n =* 1)	0	Open plating, united	3	100%	
Liu et al. [34]	2020	Combined data (*n =* 282)	282	Femur and tibia 6.6 cm (mean), (3–14), combine bifocal and trifocal	254 days SLT, 230 days DLT	EF time 385 days SLT, 341 day DLT, HI 67 days/cm SLT, 38 days/cm DLT	173	61%	Soft tissue invagination (*n =* 63), docking sites opened and refreshed docking site non-union (*n =* 29), non-union (*n =* 5), refracture (*n =* 12)	0	Refreshing of docking site (*n =* 63), bone grafting (*n =* 29), internal fixation (*n =* 12), management of non-union not reported	277	98%	
Sigmund et al. [35]	2020	ASR/bone transport (*n =* 47)	47	5.1cm	N/A	ASR—EF time 268 days, HI 60 days/cm BT—EF time 313, HI 55 days/cm	20	43%	27 unplanned operations to docking site, 13 prior to fixator removal and 14 after	14	Prior—bone grafting (4), freshening of docking site (3), docking site realignment (4), BMP (2) After—plate only (4), plating and bone grafting (2), plating and BMP (1), EF reapplication (2), intramedullary nail (5)	47	100%	1.66
Hamiti et al. [22]	2022	First stage using induced membrane followed by second stage using trifocal bone transport technique. All monolateral EF (*n =* 18)	18	6.8cm	186.4 days	HI 37.1 days/cm	14	78%	Docking site non-union (*n =* 4)	0	Autologous bone graft (*n =* 4)	18	100%	
Huang et al. [36]	2022	Total treated (*n =* 68) ASR (*n =* 32), antibiotic calcium sulphate–loaded bone transport (ACSBT) (*n =* 36)	68	12.7 cm	N/A	EF time 189 days	59	87%	Docking site non-union 9 (4 in ASR, 5 in ACSBT)	0	Internal fixation and autologous bone graft (*n =* 9)	68	100%	1.68
Huang et al. [37]	2021	Monofocal/bifocal (*n =* 85), antibiotic calcium sulphate–loaded hybrid transport with internal fixation (ACSLHT) (*n =* 44) (excluded), traditional Ilizarov bone transport (TIBT) (*n =* 41)	41	13.1 cm		EF time 432 days	32	78%	Docking site non-union 12	0	Autologous bone graft (*n =* 8), bone graft and internal fixation (*n =* 4)	44	107%	4.00

### Other factors associated with docking site outcome

**Fig. 3:**
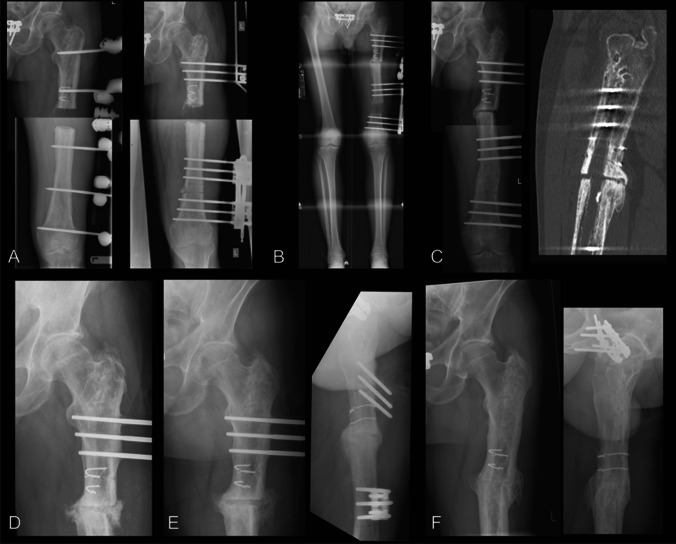
Docking site non-union in patient treated for infected non-union of femur managed by bone grafting. **A** Initial radiographs following removal of femoral nail and debridement (left). A monolateral external fixator is applied for bifocal bone transport with partial shortening (right). **B** Standing alignment films show transport and re-lengthening is complete. **C** Distraction segment has consolidated but docking site appears to only have tenuous union at best. Simple compression has been applied follow by compression and distraction (accordian technique). CT confirms docking site non-union. **D** Patient undergoes docking site procedure with freshening of the bone ends and application of autologous bone graft along with bone marrow aspirate concentrate and bioactive glass graft expander. **E** At 4 months post grafting, the docking site appears to have united, and the fixator is removed. **F** Radiographs at 12 months post fixator removal show union and remodelling of the docking site

The heterogenous nature of the studies and problems with reporting made it difficult to draw conclusions regarding what type of docking procedure is most effective. All studies in the planned procedure group included refreshing the bone ends in their operative protocol for the docking site intervention, though some undertook this using minimally invasive techniques [25, 27]. Many used autologous bone graft, though this was used selectively within studies, making any attempt to understand the effectiveness of this intervention compared to others prone to selection bias. In several studies, it was not possible to determine the outcome of different patients based upon which intervention was applied, as this was not part of the main research question and therefore not reported. Considering studies where it was possible to reliably extract relevant data, in patients where the bone graft was applied, union occurred without further intervention in 90 of 105 cases (86%) compared with 58 of 59 cases (98%) where this was not used (Fisher’s exact test *p* < 0.01). For the reasons stated above, this should be interpreted with caution. We present a case of docking site non-union in a patient treated for infected non-union of the femur managed by bone grafting in Fig. 3.

Due to the nature of the intervention, all patients in the planned intervention group were treated by bone transport, whereas some in the observed group underwent acute shortening and re-lengthening (ASR) [33, 35, 37, 38, 41]. Outcomes were therefore compared only for those patients treated without routine docking site intervention (observed group) between those managed by ASR or bone transport. In papers, where it was possible to reliably extract data, the uncomplicated docking site union rate in patients treated with ASR was 96 of 128 (75%) compared to 420 of 618 (68%) managed by bone transport. This difference was not statistically significant (chi-squared *p* = 0.11).

Some studies managed patients by bifocal and some by trifocal bone transport. In several studies, a mix of these techniques was used, and in several, it was not possible to determine outcomes by this factor due to the nature of reporting. In the remaining papers, docking site union was compared by this factor in the observed group, where it likely has the most impact. The rate of uncomplicated docking site union was 209 of 280 in those managed by bifocal transport (75%) compared to 31 of 37 managed by trifocal transport (84%). This result was not statistically significant (Fisher’s exact *p* = 0.31), though due to the issues stated this is difficult to interpret and the numbers small.

Two studies in the observed group reported the use of either poly-methyl methacrylate or calcium sulphate bone cement within the defect (54 patients) [22, 37]. The rationale for this is to induce a biologically active membrane to assist union and regenerate formation. These patients had a higher spontaneous docking site union rate when compared with the remaining 692 patients from the observed group (45 of 54 (83%) vs. 420 of 692 (61%), chi-squared test *p* = 0.0009). Again, given the heterogenous nature of the studies, this result should be viewed with caution.

### Results of narrative review

Docking site union is likely to occur by a combination of endochondral and intramembranous ossification, and the exact mechanism remains unclear [7]. As for bone healing in other situations, the interaction of host biology, local and systemic and mechanics will influence this. The following factors, relevant to the surgical technique, are thought likely to be particularly important in docking site union [7, 9, 24, 29].

**Fig. 4:**
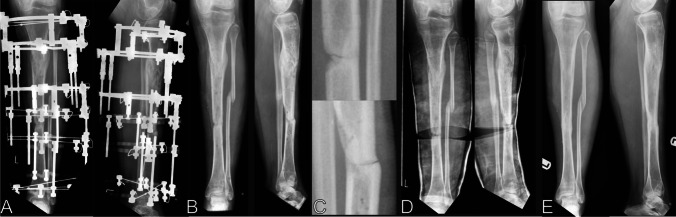
Docking site refracture managed non-operatively. **A** Patient has apparently successfully completed treatment for a Gustilo and Anderson IIIB open tibial fracture with bone loss. Note consolidated proximal transport segment and mid-diaphyseal docking site which appears united after simple compression. **B** Radiographs early after frame removal appear to show a united tibia. **C** Patient stumbles and suffers a low energy fracture. Radiographs reveal a minimally displaced refracture at the docking site. **D** A plaster cast is applied and wedged to restore alignment. **E** Spontaneous union of the refracture occurs over 4 months. Note that the docking site has better union than prior to the refracture

1. *Bone contact*: Greater contact area between bone surfaces will increase the probability of union and decrease rates of refracture. Bone contact is influenced by the geometry of the bone cuts at debridement and the accuracy of the bone transport device in maintaining alignment. We present a case of docking site refracture managed non-operatively in Fig. 4.
2. *Infection*: Unresolved infection at a docking site will affect local biology and is likely to be detrimental to union. Appropriate debridement and antimicrobial strategies likely influence this.
3. *Vascularity*: Sufficient blood supply is critical to bone healing. Thorough debridement of necrotic bone is likely to be important in achieving vascularized bony surfaces to facilitate healing.
4. *Mechanics*: Alignment and stability are important in generating appropriate mechanical environments for bony healing. This factor is affected by the stabilisation technique employed for the docking site and will also be influenced by the degree of contact.

Docking site healing is substantially different from fracture healing. In all circumstances, the local biology at the docking site will have been significantly damaged by trauma, surgery and in some cases infection. This will result in decreased local blood supply, deleterious to healing. The haematoma and inflammation, which constitute the first stage of secondary bone healing, are missing in cases of bone transport because docking occurs several weeks after the injury [43]. This results in the formation of a fibrous connective cap, again believed to have an adverse effect on bone healing. Though the bone ends are fashioned so that there should be good coaptation on contact, this is difficult to achieve, and during the transport process, bone resorption often occurs, worsening contact further [17]. The lack of fracture haematoma at docking will exacerbate this problem, potentially making gap healing less likely to occur. Even in cases of acute shortening, surgical intervention removes this haematoma to a significant extent, and the damage to the local blood supply may still result in bone resorption and problems with bone contact, with similar results [44].

Docking site operations are not without the potential for complication. The most commonly reported are local infections, soft tissue problems, and donor site morbidity [15, 25, 26]. Undertaking open surgical procedures within the confines of a circular frame can be challenging and raises issues around the maintenance of a sterile field. This leads to concerns over deep infection threatening the ultimate outcome. Docking procedures also do not guarantee union. The studies considered in the pooled analysis report rates of further docking site problems requiring intervention 0 to 32% (10% overall) in the routine intervention group. This should be considered when consenting patients for these procedures.

#### Location of the defect

The majority of included studies deal with tibial bone loss [8, 12, 14, 15, 22–28, 31–35, 37–40]. This is unsurprising since the tibia is a subcutaneous bone, prone to open injuries, with a tenuous blood supply particularly in the distal third [45]. Although many studies subdivide tibial defects into defects of the proximal, middle, and distal third, no data were identified regarding an association between docking site union rate and location within the bone. In addition, insufficient data was available to assess if the location of the corticotomy and therefore direction of the transport (antegrade/retrograde) have an influence on docking site union. Studies reporting outcomes following distraction osteogenesis in the femur are less common [41, 42]. The femur is surrounded by muscle and has a better blood supply than the tibia, meaning post-traumatic bone loss and fracture-related infections are less common. Femoral cases are more usually undertaken using monolateral fixators, due to the impracticalities of using circular fixation in the thigh. There may also be a propensity to use other approaches in the femur, such as the induced membrane technique with internal fixation, for the same reasons. Indeed, the use of magnetic lengthening nails is increasing in the femur, particularly for lengthening in cases of congenital shortening, which is not considered here. One might expect a higher docking site union rate in the femur than the tibia due to the better blood supply, but this could not be demonstrated by the data presented here.

Due to perceived differences in the relevant pathologies and outcomes, upper limb defects were not considered in the pooled data analysis.

#### Bone transport over internal implants/all-inside bone transports

In an attempt to reduce fixator time, or avoid external fixation at all, various studies have investigated a combination of internal fixation with either distraction osteogenesis by external fixation or using a motorised transport nail [19, 21]. The combination of prolonged external fixation with internal fixation in the same segment raises significant concerns about deep infection, which is potentially devastating, particularly in this context [8, 19]. The complexity and variety of the techniques employed make the impact on docking site union difficult to interpret, with some protocols including routine plating or grafting of the docking site. Studies utilising internal fixation implants routinely as part of their surgical protocol were excluded from the pooled analysis.

## Discussion

This review demonstrates that the management of docking sites remains a contentious issue, with a wide divergence of opinion on treatment protocols and ongoing research activity. Pooled analysis revealed an association in the literature between routine docking site intervention of any type and uneventful union. In those patients treated in this manner, 90% went on to union without further intervention, compared to 66% in patients where docking site procedures were only undertaken if spontaneous union did not occur.

Concerns regarding the risk of catastrophic complications, such as deep infection, threatening final treatment outcome following docking site exploration appear unfounded. Similar proportions of patients in each group went on to achieve union at the end of treatment in each group (99% in both). By the same logic, concerns that union may not be achieved as readily where docking site interventions are only undertaken when union does not arise spontaneously following simple compression are not supported by this data.

Whilst these findings initially appear to support the view that routine docking site intervention results in improved outcomes, one should consider that the mean number of procedures to attain union in the planned intervention group was higher than in the observed group (3.8 procedures per patient compared with 2.2), and this has implications for resource use and patient experience. The overall treatment time between the groups was similar. If we consider that 66% of patients in the observed group went on to spontaneous union without intervention at a similar time, then subjecting these patients to routine docking site procedures appears unnecessary. Adopting a watchful waiting approach might therefore be reasonable, carefully counselling the patient that around 1/3 of patients require further surgery to achieve union and undertaking this in a timely manner when required. Identifying patients at higher risk of non-union early for intervention would be helpful, and therefore, developing a protocol with evidence-based triggers for intervention would be a logical step. The most frequently reported risk factors for docking site non-union in the literature are shown in Table 4. Further research to explore these and develop a system to identify those at risk of non-union for planned intervention appears warranted.

**Table 4:** Proposed risk factors for docking site non-union

5,11,12,17,18,21,22,25,30,36,41,42,47,49	
1. Prolonged time to docking (> 6 months)	
2. Large defect size (> 5 cm)	
3. Reduced viability of the docked ends	
4. Poor bone contact	
5. Infection at the docking area	
6. Poor mechanical stability	
7. Soft tissue interposition (e.g., skin invagination)	
8. Microvascular dysfunction (e.g., diabetes, nicotine)	
9. Multiple previous procedures	
10. Soft tissue reconstruction (or not)	

This study is subject to limitations which should be considered when interpreting the results and applying them to clinical practice. The quality of reporting in the included studies was variable, and in a small number, it was not possible to extract sufficient data pertaining to the main outcome for inclusion. The proportion where this was not possible for secondary outcomes such as the number of procedures undertaken to union increased, resulting in missing data and smaller numbers of patients being considered. Where interventions of interest were included in a study, this was often in combination with others, making the effect of specific treatment strategies difficult to differentiate. The results of the pooled analysis are subject to all the shortcomings of the individual papers and therefore should be interpreted with caution. Though all the papers included segmental bone defects in the lower limb, these resulted from different pathologies. The treatment protocols were highly divergent between studies particularly with regard to the type of docking site intervention. Few studies were randomised, and particular cases will have been selected for different interventions for specific reasons, leading to potential selection bias. Furthermore, the outcome considered is subjective in that the decision to intervene due to the lack of progress at the docking site is taken by the treating surgeon usually without specific criteria. This means that patients in one group or the other may be managed differently, leading to these observed differences in outcome. As for the data as a whole, analysis of this nature is prone to significant confounding given the differences in patient groups from the different studies and differences in their management. It is not possible to undertake rigorous statistical analysis of the pooled continuous data, such as time to union and number of procedures, as presented in these papers. The results as presented here should therefore be taken at face value and interpreted with caution.

## Conclusions

Based on the current literature, routine intervention at docking sites may be unwarranted, potentially leading to unnecessary intervention in two thirds of patients. Regardless of the location and the bone transport technique used, a timely selective intervention in those at high risk or after a defined period of observation would appear to be a logical approach. Patient preference should also be considered. Due to the heterogeneous nature of the studies, further research is needed to better understand risk factors for non-union.
